# The role of the Notch signalling pathway in regulating the balance between neuronal and nonneuronal cells in sympathetic ganglia and the adrenal gland

**DOI:** 10.1371/journal.pone.0281486

**Published:** 2023-02-16

**Authors:** Stella Shtukmaster, Katrin Huber

**Affiliations:** 1 Department of Anatomy Institute for Anatomy and Cell Biology, University of Marburg, Marburg, Hessen, Germany; 2 Department of Medicine, University of Fribourg, Fribourg, Switzerland; University of Colorado Boulder, UNITED STATES

## Abstract

Sympathetic neurons and endocrine chromaffin cells of the adrenal medulla are catecholaminergic cells that derive from the neural crest. According to the classic model, they develop from a common sympathoadrenal (SA) progenitor that has the ability to differentiate into both sympathetic neurons and chromaffin cells depending on signals provided by their final environment. Our previous data revealed that a single premigratory neural crest cell can give rise to both sympathetic neurons and chromaffin cells, indicating that the fate decision between these cell types occurs after delamination. A more recent study demonstrated that at least half of chromaffin cells arise from a later contribution by Schwann cell precursors. Since Notch signalling is known to be implicated in the regulation of cell fate decisions, we investigated the early role of Notch signalling in regulating the development of neuronal and non-neuronal SA cells within sympathetic ganglia and the adrenal gland. To this end, we implemented both gain and loss of function approaches. Electroporation of premigratory neural crest cells with plasmids encoding Notch inhibitors revealed an elevation in the number of SA cells expressing the catecholaminergic enzyme tyrosine-hydroxylase, with a concomitant reduction in the number of cells expressing the glial marker P0 in both sympathetic ganglia and adrenal gland. As expected, gain of Notch function had the opposite effect. Numbers of neuronal and non-neuronal SA cells were affected differently by Notch inhibition depending on the time of its onset. Together our data show that Notch signalling can regulate the ratio of glial cells, neuronal SA cells and nonneuronal SA cells in both sympathetic ganglia and the adrenal gland.

## Introduction

The neural crest is a transient embryonic structure of vertebrates that issues stem cell-like progenitors. Following a process of epithelial-to-mesenchymal transition, they migrate throughout the embryo to give rise to a large array of different cells types, including bones of the skull, melanocytes, most neuronal and all glial cells of the peripheral nervous system as well as the endocrine chromaffin cells of the adrenal medulla [1–3]. How this cell diversity is generated is one of the intriguing questions of developmental biology. In principle, the fate of a neural crest cell is influenced by extrinsic and intrinsic signals that act before, during and after its migration [4–6].

The endocrine chromaffin cells of the adrenal medulla are adrenaline or noradrenaline secreting cells which share many characteristics with sympathetic neurons of the peripheral autonomic nervous system, including their origin from the neural crest and their expression of the catecholaminergic marker enzymes tyrosine-hydroxylase (TH) and Dopamine-β-Hydroxylase (DBH) [1, 3]. Unlike chromaffin cells, sympathetic neurons extent long neurites and express typical neuronal markers, i.e. neurofilament and SCG10 [3, 7]. While chromogranin A [8] and Phenylethanolamine-N-Methyltransferase (PNMT) [1], which is responsible for adrenaline synthesis, are considered as typical chromaffin cell markers, both can also be detected in other sympathetic structures [9, 10].

It was originally believed that sympathetic neurons and adrenal chromaffin cells develop from a common noradrenergic sympathoadrenal (SA) progenitor that segregates into neuronal or endocrine cells [11, 12]. A later genetic tracing analysis in mice starting at E11.5 revealed that at least half of all chromaffin cells analyzed at E17.5 are generated from nerve-associated Schwann cell precursors (SCP) [13].

Likewise, the majority of chromaffin cells that colonize the Zuckerkandl Organ (ZO) as well as several sympathetic neurons at the posterior-most level of the dorsal aorta may also derive from glial progenitors [14]. The above studies might relate to a slightly later contribution of neural crest-derived Schwann cell progenitors that is primarily directed to the formation of adrenomedullary cells. In fact, early electroporation of premigratory crest cells *in ovo* showed that individual progenitors can give rise to both chromaffin cells and sympathetic neurons, suggesting that the fate of these cells is determined after delamination [15]. However, the mechanisms and the timing underlying the above segregation remain unclear.

Notch signalling is a well-known regulator of fate decisions [16]. Notch receptors are transmembrane proteins that are activated by members of the Delta/Serrate/Jagged-2 (DSL) Family [17–20]. Interaction of Notch receptors with their ligands leads to γ-secretase dependent cleavage of the Notch intracellular domain (ICD) from the membrane and its translocation to the cell nucleus. Inside the nucleus it activates Notch downstream targets including transcriptional repressors of the hairy enhancer of split (Hes) family, which have been shown to downregulate the expression of proneural genes in the developing nervous system [21–23]. Several modulators of the Notch signalling pathway have been identified. Numb, a membrane associated molecule, asymmetrically segregates in dividing cells and antagonizes Notch-mediated lateral inhibition via interaction with ICD leading to its degradation [24–28]. Suppressor of Hairless also known as CSL (CBF-1/Su(H)/Lag-1) is a DNA-binding protein that is the main intracellular activator of the Notch signalling pathway [29]. Following interaction with Notch ICD, Su(H) carries Notch signal into the nucleus and activates the *Enhancer of split complex of genes* that suppresses neural development [30, 31]. Dominant-negative Suppressor of Hairless (dnSu(H)) has been demonstrated to inhibit Notch signalling in ventral mesoderm [32], and in sympathetic and sensory ganglia [33].

Furthermore, Mastermind (MAML) is an essential nuclear element that plays a crucial role in the activity of Notch signalling pathway [34, 35]. Recent studies showed that MAML is a nuclear protein that stabilizes and forms DNA-binding complexes with Notch ICD and CSL proteins during activation of target genes such as *Enhancer of split* in *Drosophila* or *Hairy Enhancer of split* (*HES*)-1 and *HES-5* [36, 37]. Generation of a dominant negative mutant of MAML (dnMAML) showed that it is able to inhibit Notch-mediated transcriptional activation in early hematopoietic development [38]. Both dnMAML and dnSu(H) act in a complex with NICD, inhibit its transcriptional activity [39, 40] and repress Notch activity.

Notch signalling has important functions during development of the sympathetic nervous system [41]. The Notch ligand Delta like 1 (Dll1)–is a direct target of the proneural transcription factor MASH1 in developing sympathetic neurons [42]. In turn, the Notch downstream target Hes1 inhibits MASH1 expression [36]. An *in situ* hybridization analysis in chicken embryo has demonstrated that several Notch signalling pathway components such as Notch1, Dll1 and Hes5 are present in the sympathetic ganglia and the DRG at E3 and E4, and downregulated from E5 onwards [33]. In addition, Notch was shown to regulate the balance between neurogenesis and gliogenesis as well as progenitor maintenance [17, 21, 43]. Upregulation of Notch in chick NC cells decreased the neuronal population in sympathetic ganglia, whereas downregulation of Notch increased the number of neurons [33, 44].

While the early roles of Notch signalling have been well described for developing sympathetic ganglia, its role in the developing adrenal gland and possible implication in the neuronal versus chromaffin cell fate decision has not been investigated.

In the present study we examined the role of Notch in development of chromaffin cells and sympathetic neurons via gain- and loss-of-function approaches. For this purpose, we performed hemi-tube electroporations of activated Notch 1, dominant negative Su(H)–dnSu(H), Numb and dominant negative mastermind (dnMAML) into pre-migratory NC cells at the level of somites 18–24, the axial levels from which adrenal chromaffin cells and part of sympathetic neurons arise. Our data show that Notch signalling is not directly required for the decision between chromaffin cells and sympathetic neurons. Nevertheless, it regulates the balance between catecholaminergic and glial cells in both sympathetic ganglia and the adrenal gland *in ovo*.

## Methods

Fertilized white Leghorn chicken (Gallus gallus) eggs were obtained from commercial sources (Haas, France and LSL Rhein-Main, Dieburg). Embryos were staged according to Hamburger and Hamilton [45].

The following plasmids were used for hemitube electroporation: Suppressor of Hairless (Su(H)): *pmiw*-Flag-*xdnSu*(*H*) [28]; Numb: pMIW-FLAG-cNumb [28]; the Notch 1 intracellular domain: pMIW-FLAG-CNIC [28]; GFP: pCAGGS-AFP [46].

For conditional over-expression pTRE-dnMAML1-flag-IRES-GFP was co-electroporated with pCAGGS-rtTA2s-M2 (Tet on) [34]. Doxycycline (250ml of 0.1mg/μl) was administered every 12 hours in order to activate plasmid transcription.

For hemitube electroporation, two electrodes were placed at either side of the embryo. Plasmid solution at a concentration of 4μg/μl was injected into the lumen of the neural tube and hemitube electroporation was achieved by applying two electrical pulses of 10ms and voltage of 25V at 100ms intervals using an ECM^®^ 830 electroporator (BTX Harvard Apparatus). Embryos were then re-incubated until E6.

### Embryo processing and sectioning

Embryos were fixed in 4% formaldehyde overnight, washed twice in 1xPBS, and processed for paraffin (Leica Biosystems) or cryo-embedding. Paraffin and cryo blocks were cut into 10μm thin sections on a microtome (Leica) and cryostat (Leica), and sections were mounted on Superfrost slides (Langenbrinck).

### Immunohistochemistry and in situ hybridization

Sections were immunolabeled with the following antibodies for GFP (rabbit anti-GFP; 1:200 in BA; AB290, Abcam, Cambridge, UK), TH (mouse anti-TH 1:400 in BA; MAB318, Sigma-Aldrich, Germany), and P0 (chicken anti-P0 1:200 in BA, 1E8, Hybridoma Bank, USA). A Non-radioactive in situ hybridization for chick-specific neurofilament-M (NF-M) [47] was performed as described [48].

### Data analysis and statistics

The analysis of paraffin and cryo sections was performed with a Zeiss Axiophot equipped with objectives 5x, 10x and 20x, and with Zeiss Axiophot. The microscope was equipped with fluorescence and filters for GFP (green), TH, and P0 (red), DAPI (blue) and bright field for non-fluorescence staining. Images were taken with a Zeiss camera with AxioVision programme, saved in JPG or TIFF format. If necessary brightness and contrast were adjusted to the entire image using AxioVision and Photoshop 6.0 (AdobeSystems, USA), over layered images were prepared using GIMP 2.0.

To evaluate the percentages of GFP+/TH+/NF+ in SG and in AG following over-expression of dnSu(H), Numb, Notch1, and dnMAML versus control an unpaired two-tailed Student’s *t*-test was carried out. To analyze the percentages of glia cells (GFP+/P0+) in SG and AG following over-expression and down-regulation of Notch versus control an unpaired two-tailed Student’s *t*-test was performed. Statistical analysis was performed using SigmaPlot 12.0 (Systat Software GmbH, Erkrath, Germany).

## Results

### Manipulation of the Notch pathway alters the number of TH-positive and P0-positive cells in both sympathetic ganglia and adrenal gland

**Fig 1:**
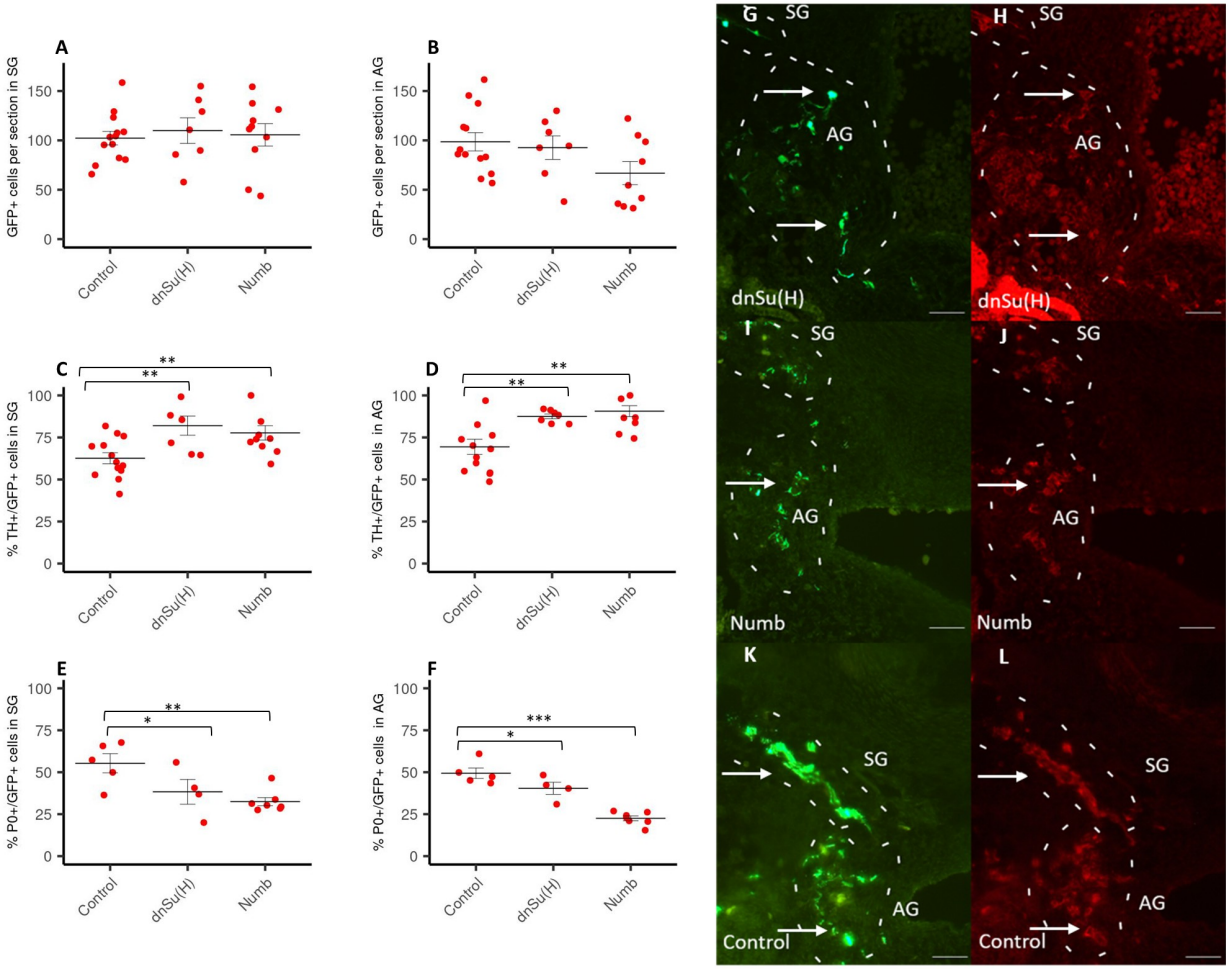
Analysis of GFP+, TH+/GFP+ and P0+/GFP+ cells in SG and AG following downregulation of Notch. Amount of GFP-positive cells per section (% of control) in sympathetic ganglia (SG) (A) and the adrenal gland (AG) (B) of E6 chick embryos after electroporation with dnSu(H) or Numb at E2. Amount of TH+/GFP+ cells (% of the total number of GFP-positive cells) in sympathetic ganglia (SG) (C) and the adrenal gland (AG) (D) of E6 chick embryos after electroporation with dnSu(H) or Numb at E2 (control n = 13, dnSu (H) n = 7, Numb n = 10). Amount of P0+/GFP+ cells in SG (E) and AG (F) of E6 chick embryos after electroporation with, dnSu(H) or Numb at E2 (control n = 5, dnSu(H) n = 4, Numb n = 7). Data are presented as mean +/- S.EM. *p≤ 0.05; **P ≤0,01; ***P ≤0,001; n: number of experimental animals. Photomicrographs showing GFP and P0-positive cells in SG and the AG (G-L) of E6 chick embryos after electroporation with dnSu(H) (G, H), Numb in (I, J) and control (K, L) at E6. Embryos were immnunostained using antibodies against GFP, (G, I, K) and P0 (H, J, L). The values used to build the graphs in S1 File. Scale bar: 100μm.

To begin exploring the role of Notch in the early development of sympathetic neurons and chromaffin cells we downregulated Notch signalling using two different inhibitors, dominant negative pMiw-dnSu (H)—dnSu(H) and Numb. Hemi-tubes were electroporated at E2 (HH13) and analyzed at E6. As shown in Fig 1 this treatment did not alter the number of GFP+ cells that reached the sympathetic ganglia (Fig 1A) or the adrenal gland (Fig 1B) when compared to control embryos.

However, we observed a significant increase in the percentage of TH-positive cells out of GFP positive cells in both AG and SG after the treatment with both inhibitors (Fig 1C and 1D).

To determine whether the amount of TH-positive cells was increased at the expense of the number of glial cells, we employed P0, an established marker for glial cells in the chicken embryo [49]. Our data show that electroporation with dnSu(H) and Numb significantly reduced the percentage of P0-positive out of GFP-positive cells in the sympathetic ganglia with both treatments. Both treatments also reduced the percentage of P0+ cells in the adrenal gland, yet only the Numb manipulation was statistically significant (Fig 1E and 1F).

**Fig 2:**
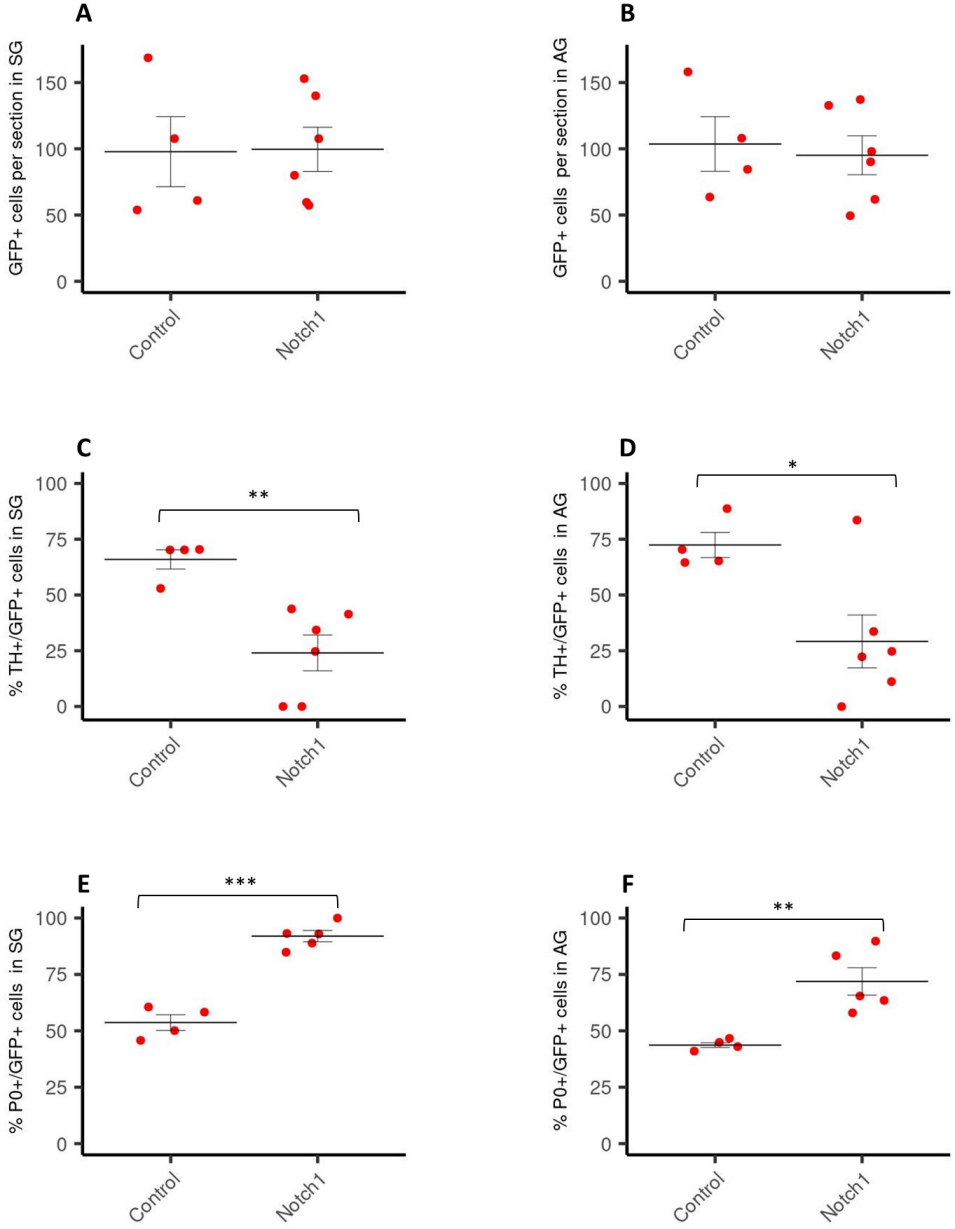
Analysis of TH+/GFP+ and P0+/GFP+ cells in SG and AG following overexpression of Notch. Number of GFP-positive cells per section (% of control) in sympathetic ganglia (SG) (A) and the adrenal gland (AG) (B) of E6 chick embryos after electroporation with Notch1 at E2. Amount of TH+/GFP+ cells (% of the total number of GFP-positive cells) in SG (C) and AG (D) of E6 chick embryos after electroporation with Notch1 at E2 (control = 4, Notch1 = 6). Amount of P0+/GFP+ cells in SG (E) and AG (F) of E6 chick embryos after electroporation with Notch1 at E2 (control n = 4, Notch1 = 5). Data are presented as mean +/- S.EM. *p≤ 0.05; **P ≤0,01; ***P ≤0,001; n: number of experimental animals. The values used to build the graphs in S2 File.

In agreement with these results, the activation of the Notch signalling pathway by hemitube-electroporation with the plasmid pMIW-FLAG-CNIC at E2, did not alter the number of GFP+ cells in SG and the AG as compared to control embryos (Fig 2A and 2B), but led to a decreased percentage of TH-positive cells and an increased percentage of P0-positive cells in both locations (Fig 2C–2F).

### Inhibition of Notch signalling differentially affects the number of neurofilament-negative and neurofilament-positive cells in a time dependent manner

**Fig 3:**
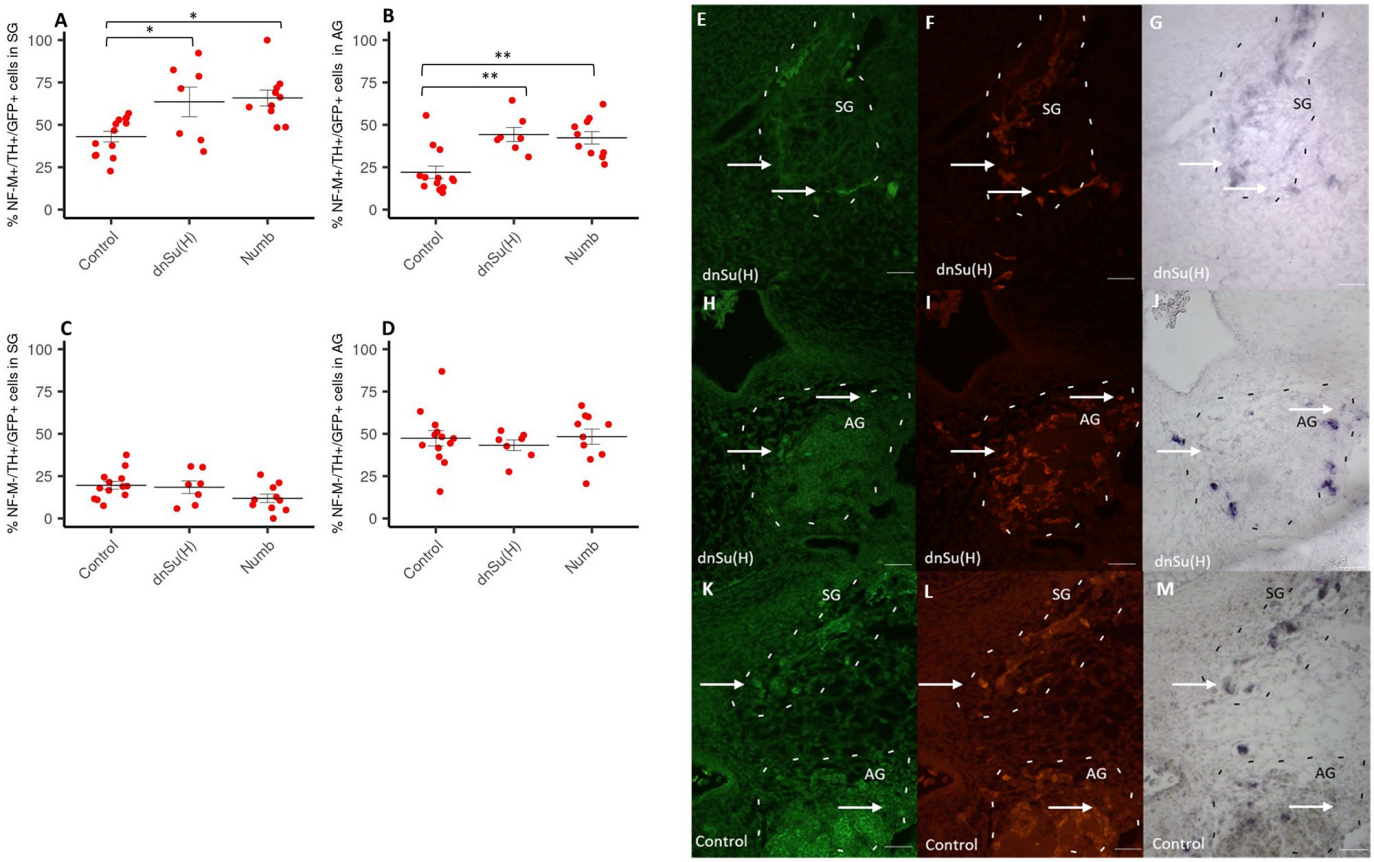
Analysis of neuronal and nonneuronal cells in SG and AG following downregulation of Notch. Amount of NF-M+/TH+/GFP+ cells in SG (A) and AG (B), and amount of NF-M-/TH+/GFP+ cells in SG (C) and AG (D) of E6 chick embryos after electroporation with dnSu(H) or Numb at E2 (control n = 13, dnSu(H) n = 7, Numb n = 10). Data are presented as mean +/- S.EM. and expressed as percentage of the total number GFP+ cells. *p≤ 0.05; **p ≤0,01; ***p ≤0,001; n: number of experimental animals. Photomicrographs showing the expression of GFP, TH and NF in sympathetic ganglia (SG) and the adrenal gland (AG) of E6 chick embryos after electroporation with dnSu(H), and control at E2. Embryos electroporated with dnSu(H): SG (E-G) and AG (H-J) using antibodies for GFP (E, H), TH (F, I) and in situ hybridization for NF-M mRNA (G, J). Embryos electroporated with control: SG and AG (K-M) at E6 using antibodies for GFP (K), TH (L) and in situ hybridization for NF-M mRNA (M). The values used to build the graphs in S1 File. Scale bar: 100μm.

To investigate whether Notch downregulation distinctly affects the generation of sympathetic neurons and chromaffin cells we employed the neuronal marker neurofilament to distinguish between sympathetic neurons and chromaffin cells. According to the literature, both sympathetic neurons and adrenal chromaffin cells are characterized by the expression of TH, however chromaffin cells lack NF, which is highly expressed in neurons [7, 12]. We detected a significant increase in the percentage of NF-M+/TH+/GFP+ cells out of total GFP+ cells in both the SG and the AG upon inhibition of Notch signalling at E2 using dnSu(H) and Numb (Fig 3A and 3B). In contrast, we did not observe any change in the percentage of NF-M-/TH+/GFP+ cells out of total GFP+ cells (Fig 3C and 3D). As shown in Fig 3E–3M, immunohistochemical and *in situ* hybridization staining for NF-M, GFP and TH; white arrows point on NF-M+/TH+/GFP+ cells in SG and AG at E6 after electroporation with dnSu(H) at E2 (Fig 3E–3J), and in Fig 3K–3M NF-M+/TH+/GFP+ cells in SG and TH+/GFP+ cells in AG after electroporation with control.

**Fig 4:**
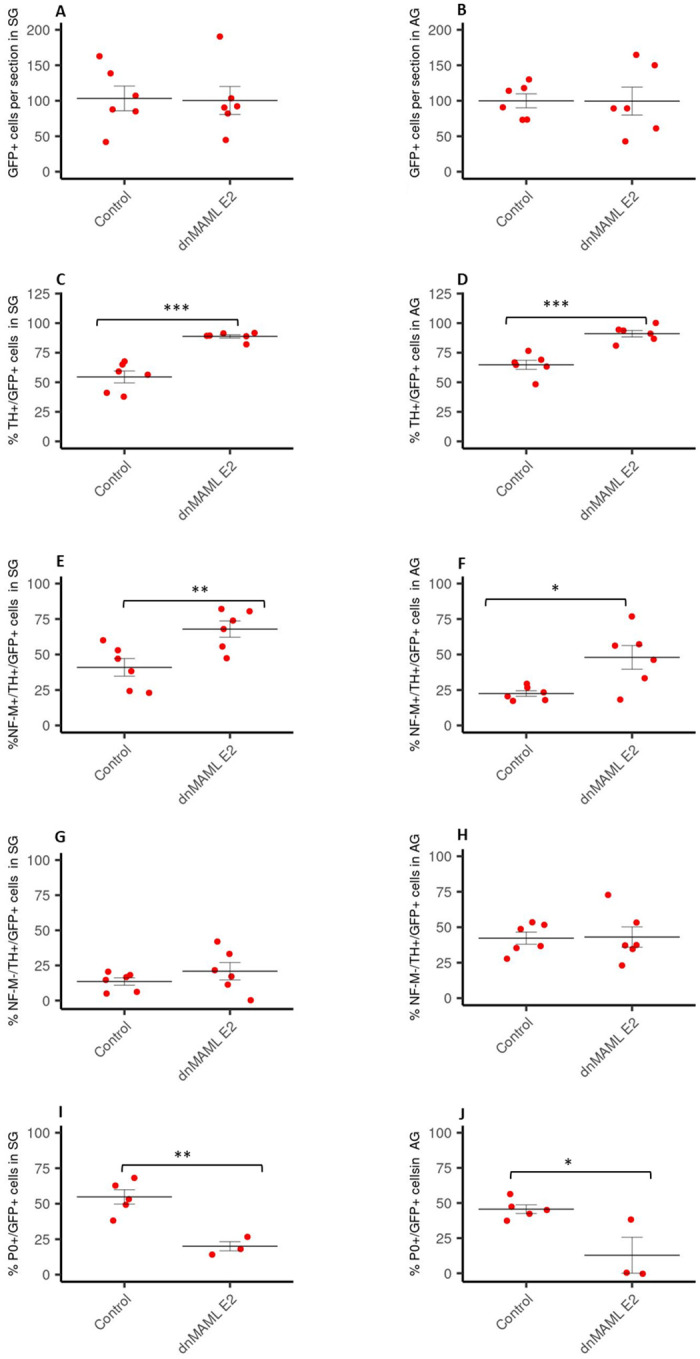
Quantification of cells expressing various markers in SG and AG following electroporation with dnMAML at E2. Number of GFP-positive cells per section (% of control) in sympathetic ganglia (SG) (A) and the adrenal gland (AG) (B) of E6 chick embryos after electroporation with dnMAML at E2 and immediate tet-on activation expressed as percentage of control experiments. Percentage of TH-positive cells out of GFP-positive cells counted in SG (C) and AG (D) of E6 chick embryos after electroporation with dnMAML at E2 and immediate tet-on activation (control n = 6, dnMAML = 6). Amount of NF-M+/TH+/GFP+ (E, F) and NF-M-/TH+/GFP+ (G, H) in SG (E, G) and AG (F, H) of E6 chick embryos after electroporation with dnMAML at E2 and immediate tet-on activation (control n = 6, dnMAML n = 6). Data are expressed as percentage of the total number of GFP-positive cells. Percentage of P0-positive cells out of GFP-positive cells in SG (I) and AG (J) of E6 chick embryos after electroporation with dnMAML at E2 and immediate tet-on activation (control n = 4–5, MAML = 3–4). Data are presented s mean +/- S.EM. *p≤ 0.05; **p ≤0,01; ***p ≤0,001; n: number of experimental animals. The values used to build the graphs in S3 File.

Next, we asked whether there is a specific time window of Notch inhibition that accounts for the observed effects. To this end, we mis-expressed a dominant negative form of mastermind (dnMAML), a specific inhibitor of Notch mediated transcription, that was subcloned into a Tet-inducible system [38, 50]. pTRE-dnMAML1-flag-IRES-GFP was co-electroporated together with pCAGGS-rtTA2s-M2 into the NT of chick embryos on embryonic day 2 (E2) HH13. To compare the effects of dnMAML-1 with those of Numb and dnSu(H), doxycycline was first added immediately after the electroporation and embryos were analyzed at E6. Consistent with the data obtained after electroporation of Numb and dnSu(H), we did not observe an alteration of the number of GFP+ cells in the adrenal gland or sympathetic ganglia following activation of dnMAML expression (Fig 4A and 4B), while the percentage of TH+/GFP+ cells (Fig 4C and 4D) and the percentage of NF-M+/TH+/GFP+ cells out of GFP+ cells was increased in both locations (Fig 4E and 4F). Further the percentage of NF-M-/TH+/ GFP+ cells out of GFP+ cells was not altered (Fig 4G and 4H), and the percentage of P0-positive cells was significantly lower in both locations (Fig 4I and 4J).

**Fig 5:**
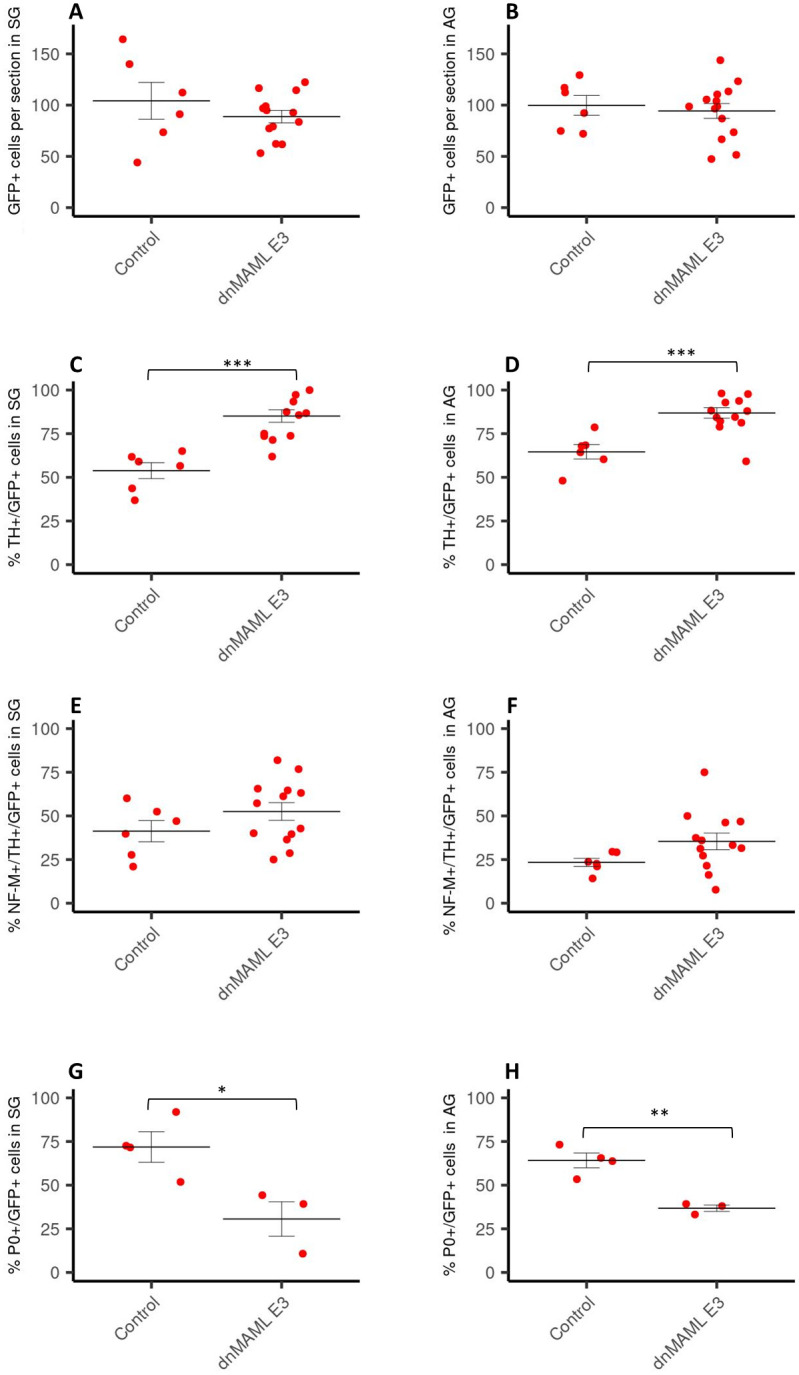
Quantification of cells expressing various markers in SG and AG following electroporation with dnMAML at E3. Amount of GFP-positive cells per section (% of control) in SG (A) and AG (B) of E6 chick embryos after electroporation with dnMAML at E2 with tet-on activation at E3 (control n = 6, dnMAML n = 13). Percentage of TH+/GFP+ cells out of GFP-positive cells in SG (C) and AG (D) of E6 chick embryos after electroporation with dnMAML at E2 with tet-on activation at E3. Amount of NF-M+/TH+/GFP+ cells in SG (E) and AG in E6 (F) chick embryos after electroporation with dnMAML at E2 with tet-on activation on E3 (control n = 6, dnMAML n = 13). Percentage of P0+/GFP+ cells out of GFP-positive cells in SG (G) and AG (H) of E6 chick embryos after electroporation with dnMAML at E2 with tet-on activation on E3 (control n = 4, dnMAML = 3). Data are presented as mean +/- S.EM. *p≤ 0.05; **p ≤0,01; ***p ≤0,001; n: number of experimental animals. The values used to build the graphs in S4 File.

In the next set of experiments, we activated the expression of dnMAML only at E3 to test the effect of Notch inhibition starting at a time point when future SA progenitors have already arrived in a position next to the dorsal aorta [3, 51]. Then, we analyzed the embryos on E6 after the formation of the adrenal gland and the sympathetic ganglia. We did not detect a difference in the number of GFP-positive cells that reached the sympathetic ganglia (Fig 5A) and the adrenal gland (Fig 5B) between control and dnMAML electroporated embryos. In accordance with the previous experiments, inhibition of Notch signalling by dnMAML from E3 onwards increased the percentage of TH+ cells out of total GFP+ in both SG and AG (Fig 5C and 5D).

Surprisingly, in contrast to the long-term (E2-6) inhibition of Notch by all treatments, the inhibition by dnMAML from E3 onwards did not result in a significant increase in the percentage of NF-M+/TH+/GFP+ cells out of total GFP+ cells in either the SG or the AG (Fig 5E and 5F). This suggests that the increase of TH+ cells may at least in part be due to an increase in nonneuronal TH+ cells, i.e. putative chromaffin cells.

Finally, consistent with previous results, the delayed Notch inhibition by dnMAML also resulted in a lower percentage of P0+ cells (Fig 5G and 5H).

Together, these results suggest that interference with the Notch signalling pathway influences the balance between neuronal and glial cells in sympathetic ganglia as well as in the adrenal gland and points to a time window for Notch-mediated neuronal specification between E2-3.

## Discussion

In this study we investigated the role of the Notch signalling pathway in development of sympathoadrenal progenitors in both adrenal gland and sympathetic ganglia. Our data show that manipulation of the Notch signalling pathway alters the ratio of TH+ cells (sympathoadrenal cells) to P0+ cells (glial precursor cells) in both locations. The number of GFP+ cells that had immigrated to the adrenal gland or the sympathetic ganglia was not affected, which is in agreement with a study in quail and mice embryos showing that gain and loss of Notch function does not alter the behavior of early NC [52].

In accordance with the literature, inhibition of the Notch signalling pathway leads to the increased numbers of TH+ cells and decreased numbers P0+, while activation of the Notch signalling pathway had the opposite effect [21]. Interestingly, TH+ cells with neuronal properties (i.e. NF+/TH+) were differentially affected depending on the time of onset of Notch inhibition. Early onset of Notch inhibition in premigratory neural crest cells at E2 led to an increase of neuronal-like TH+ cells in sympathetic ganglia as well as the adrenal gland, while the number of non-neuronal TH+ cells, i.e. putative chromaffin cells, was not significantly altered. Similar results were observed with all three methods of Notch inhibition employed in this study, i.e. Numb, dnSu(H) and activation of dnMAML at E2. In contrast, onset of Notch inhibition at E3 lead to an increase of TH+ cells in both sympathetic ganglia and the adrenal gland without significantly increasing the numbers of neuronal-like TH+ cells. This suggests that the increase of TH+ cells is at least partly due to an increase of NF-/TH+ cells in both the adrenal gland and also sympathetic ganglia. It should be noted that significant amounts of neurofilament-negative cells have been found in normal chick sympathetic ganglia. These cells likely represent chromaffin-like small intensively fluorescent cells (SIF) cells [3]. Likewise, sympathetic neurons have been detected within the adrenal gland [53].

Our data show that early inhibition of Notch-signaling can shift the ratio between neuronal and nonneuronal SA cells in sympathetic ganglia and the adrenal gland. However, the fact that early Notch inhibition resulted in increased numbers of NF+/TH+ cells at the expense of P0+ cells without diminishing the numbers of NF-/TH+ cells in the adrenal gland or sympathetic ganglia makes it unlikely that Notch signalling can directly influence the differentiation of sympathetic neurons versus that of chromaffin cells. Rather, it confirms the role of Notch in the sympathetic neuron versus glial cell fate decision which has been described before for sensory and sympathetic ganglia [21, 33] and suggests similar effects within the adrenal gland. The data obtained following early Notch-inhibition might suggest that the generation of chromaffin cells, unlike that of sympathetic neurons, is not modulated by Notch signalling.

However, the lack of effect on the numbers of non-neuronal TH+ cells that was observed upon early Notch inhibition may well be the result of two opposing effects at different time windows. In addition to the classic dogma that postulated the existence of common TH+ sympathoadrenal progenitors that differentiate into NF- chromaffin cells or NF+ neuronal cells depending on their final environment [54], it was later suggested that at least a half of chromaffin cells are derived from Schwann/glial progenitors [13]. According to our previous data, sympathetic neurons and chromaffin cells arise from common progenitors at the neural tube level, suggesting that diversification of these progenitors occurs after delamination from the neural tube [15]. Thus, Notch signalling may be indirectly involved in the regulation of this diversification by its putative function on the maintenance of SCPs, which later give rise to chromaffin cells.

Our data are consistent with the hypothesis that Notch signalling may play sequential roles in the differentiation of chromaffin cells. Early inhibition of Notch may reduce the generation of chromaffin cells by reducing the maintenance of SCPs, while later inhibition may enhance it by promoting catecholaminergic cell differentiation.

Together, our findings confirm the role of Notch signalling in regulating the ratio of sympathoadrenal to glial cells in sympathetic ganglia and in addition demonstrate the same for the adrenal gland. As expected, our data do not point to a direct role of Notch signalling in chromaffin cell versus sympathetic neuron differentiation. However, Notch signalling may modulate the ratio of neuronal to non-neuronal TH+ cells in the adrenal gland and sympathetic ganglia depending on its precise timing of activity.

## Supporting information

S1 File Values used to build the graphs for dnSu(H) and Numb. (XLSX)

S2 File Values used to build the graphs for Notch1. (XLSX)

S3 File Values used to build the graphs for dnMAMLE2. (XLSX)

S4 File Values used to build the graphs for dnMAMLE3. (XLSX)
